# Transpapillary biliary drainage using a forward-viewing endoscope for distal malignant biliary obstruction after placement of a duodenal stent for type I duodenal stenosis

**DOI:** 10.1055/a-2271-6994

**Published:** 2024-03-14

**Authors:** Yuichi Hirata, Takahiro Oribe, Kosuke Sugihara, Mizuka Yonezawa, Daisuke Orita, Yoshihiro Okabe

**Affiliations:** 1 469536 Department of Gastroenterology, Kakogawa Central City Hospital, Kakogawa, Japan

Transpapillary biliary drainage using a side-viewing duodenoscope is widely performed for patients with obstructive jaundice. In patients with pancreaticobiliary cancer, both distal malignant biliary obstruction (MBO) and duodenal stenosis may be complicated ( Fig. 1 ) 1 , and it is often difficult to insert the duodenoscope in patients with duodenal stenosis on the oral side of the major papilla, especially. Endoscopic ultrasound-guided biliary drainage and percutaneous transhepatic biliary drainage are useful as alternative procedures; however, the presence of ascites or collateral flow makes it impossible to perform these procedures. We report a case of transpapillary biliary drainage for MBO using a forward-viewing endoscope after placement of a duodenal stent for type I duodenal stenosis.

**Fig. 1:**
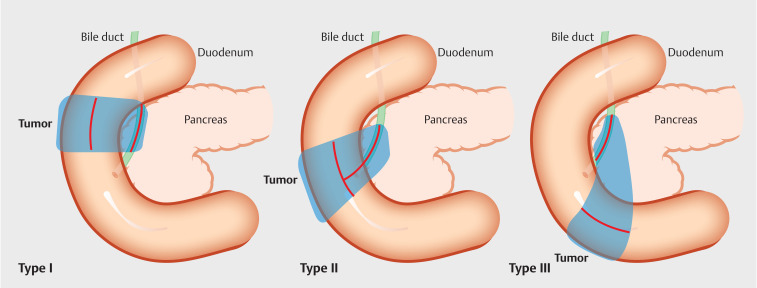
Schematic representations of duodenal stenosis. Three types of duodenal stenosis with biliary stenosis are shown, classified according to the anatomical location of the duodenal stenosis in relation to the major papilla. Blue areas indicate tumors and red lines indicate stenoses.

A 68-year-old man with pancreatic cancer was admitted to our institution because of high fever. Blood tests showed obstructive jaundice, and computed tomography (CT) revealed biliary dilatation despite the presence of endoscopic biliary drainage ( Fig. 2 **a** ). CT also revealed ascites ( Fig. 2 **b** ) and collateral flow between the stomach and the liver because the extrahepatic portal vein was occluded by tumor invasion ( Fig. 2 **c** ). Although endoscopic retrograde cholangiopancreatography (ERCP) using a side-viewing duodenoscope (TJF-Q290V; Olympus Medical Systems, Tokyo, Japan) was attempted, it was difficult to insert the duodenoscope into the second portion of the duodenum because of type I duodenal stenosis ( Fig. 3 **a** ). Therefore, a duodenal stent was placed ( Fig. 3 **b** ). Although the duodenoscope was inserted into the second portion through the duodenal stent, ERCP could still not be performed due to poor view ( Fig. 4 **a** ). The major papilla could be visualized by flipping up the anal end of the duodenal stent using a forward-viewing endoscope (SIF-H290S; Olympus Medical Systems, Tokyo, Japan). Biliary cannulation was then performed ( Fig. 4 **b** ), and a fully covered self-expandable metal stent was placed for the MBO ( Fig. 4 **c** , Video 1 ).

**Fig. 2:**
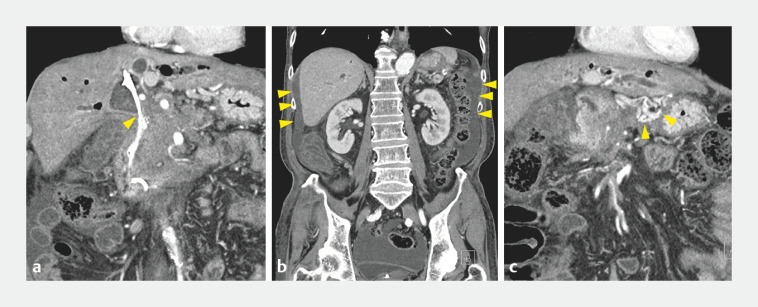
Computed tomography images. **a** Biliary dilatation (arrowhead). **b** Ascites (arrowheads). **c** Collateral flow between stomach and liver (arrowheads).

**Fig. 3:**
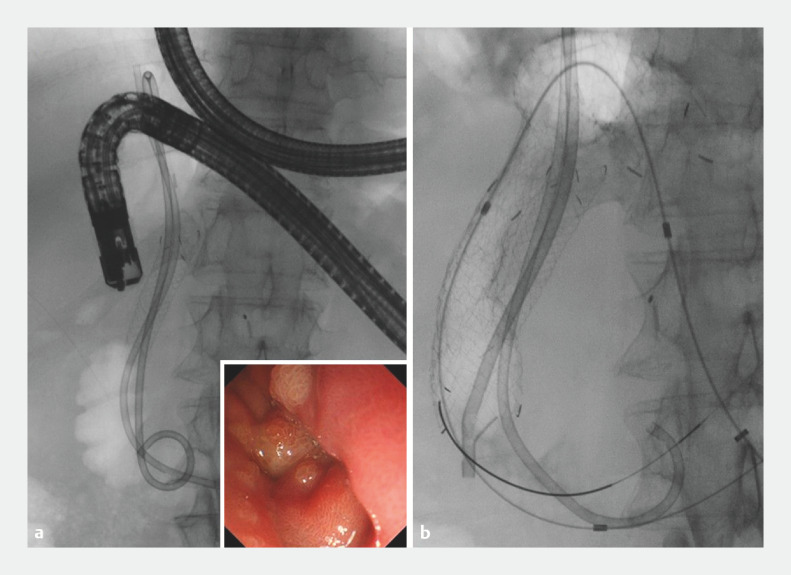
Fluoroscopic images. **a** It was difficult to insert the scope into the second portion of the duodenum due to duodenal stenosis of type I. **b** A duodenal stent was placed.

**Fig. 4:**
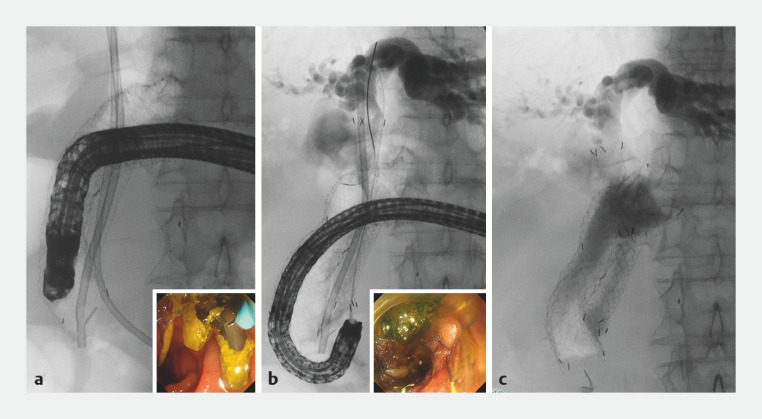
Fluoroscopic images. **a** Although a side-viewing duodenoscope was inserted into the second portion, endoscopic retrograde cholangiopancreatography could still not be performed due to poor view. **b** Biliary cannulation was performed by flipping up the anal end of the duodenal stent using a forward-viewing endoscope. **c** A fully covered self-expandable metal stent was placed.

Transpapillary biliary drainage using a forward-viewing endoscope for distal malignant biliary obstruction after placement of duodenal stent is useful for patients with type I duodenal stenosis.Video 1

Transpapillary biliary drainage for MBO using a forward-viewing endoscope after placement of a duodenal stent is useful for patients with type I duodenal stenosis, ascites, and collateral flow.

Endoscopy_UCTN_Code_TTT_1AR_2AZ

## Notes

Endoscopy E-Videos https://eref.thieme.de/e-videos
*E-Videos* is an open access online section of the journal *Endoscopy* , reporting on interesting cases and new techniques in gastroenterological endoscopy. All papers include a high-quality video and are published with a Creative Commons CC-BY license. Endoscopy E-Videos qualify for HINARI discounts and waivers and eligibility is automatically checked during the submission process. We grant 100% waivers to articles whose corresponding authors are based in Group A countries and 50% waivers to those who are based in Group B countries as classified by Research4Life (see: https://www.research4life.org/access/eligibility/ ). This section has its own submission website at https://mc.manuscriptcentral.com/e-videos .
